# Unexplained Hypoxemia in School-Age Child: Do Not Forget the Double Superior Vena Cava

**DOI:** 10.3390/children9091272

**Published:** 2022-08-24

**Authors:** Luca Pecoraro, Enrico Boninsegna, Emilio Simonini, Paolo Francia, Stefano Colopi, Angelo Pietrobelli

**Affiliations:** 1 Pediatric Unit, Department of Surgical Sciences, Dentistry, Gynecology and Pediatrics, University of Verona, 37129 Verona, Italy; 2 Department of Medicine, University of Verona, 37129 Verona, Italy; 3 Department of Radiology, Azienda Socio Sanitaria Territoriale di Mantova, 46100 Mantova, Italy; 4 Pennington Biomedical Research Centre, Baton Rouge, LA 70808, USA

**Keywords:** persistent left superior vena cava, child, oxygen saturation, vascular malformation, tomography

## Abstract

Persistent left superior vena cava (SVC) is a rare congenital malformation of the thoracic venous system. We report a case involving a 7-year-old boy, who was admitted to our CT scanning room because of an incidental discovery of low blood-oxygen levels (90–94% in good health). A persistent left SVC was depicted, with drainage in the left atrium and a resultant right-to-left shunt;right SVC was present, draining to the right atrium. A small bridging vein was depicted. A comprehensive cardiological assessment with echocardiography was performed, but no other anomalies were found. He was successfully treated with a percutaneous endovascular approach and vascular plug deployment. A complete occlusion of the left SVC was obtained, with normalization of the oxygen saturation. Persistent left SVC is a rare vascular anomaly, often incidentally detected. Physicians should be aware because it may have significant clinical implications, especially during catheterization procedures or when associated with other cardiac malformations.

## 1. Introduction

Persistent left superior vena cava (SVC) is a rare congenital malformation of the thoracic venous system [1,2]. The prevalence is estimated between 0.2 to 0.5% in the general population, but it is significantly more common in patients affected by congenital heart disease, with a prevalence of 4.5% [1,2,3]. In most cases it drains into the right atrium, with no symptoms, and is detected when cardiovascular imaging is performed for unrelated reasons [3,4]. Rarely left SVC drains into the left atrium, causing a right-to-left shunt [5]. We report the case of a young boy with chronic hypoxemia caused by persistent left SVC, successfully treated with a percutaneous endovascular approach.

## 2. Case Report

**Figure 1:**
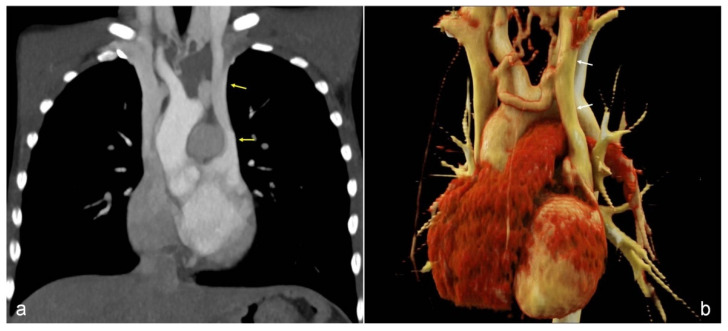
(**a**). Computed tomography reveals a persistent left SVC (arrows) with drainage in the left atrium and resulting right-to-left shunt; right SVC is regularly present, draining to the right atrium.(**b**). A3D-volume rendering image of the computed tomography confirms the condition; it allows to better appreciate a bridging vein between left and right SVS, with mean diameter of 7 mm.

**Figure 2:**
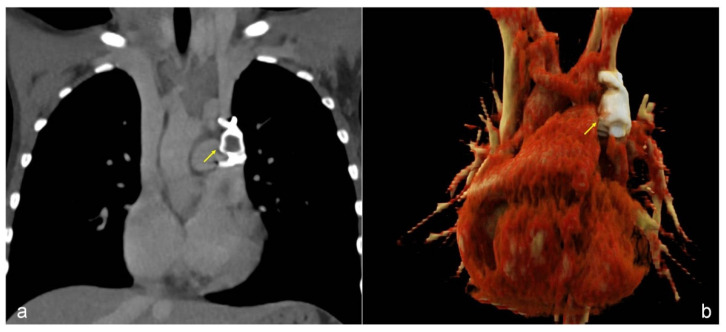
(**a**). Computed tomography two months after occlusive device insertion (arrow). (**b**). A 3D-volume rendering image shows proper position of the plug and confirms left SVC occlusion. Blood reaches the right SVC through the bridging vein; its current diameter is 9 mm.

A 7-year-old boy was referred to our attention because of occasional fatigue during sport activity and persistent presentation of low blood-oxygen levels (90–94% in good health) both at rest and under load during clinic evaluation. Physical examination was normal and laboratory tests were not specific. He was not suffering from allergies. Chest X-ray and pulmonary function testing were normal. Based on these symptoms and low blood-oxygen level, a diagnosis of asthma was hypothesized, and an anti-asthmatic treatment was initiated without significant improvement of blood-oxygen level. Then, he was admitted to our Computed Tomography (CT) scanning room and we examined the chest with a children-dedicated protocol: low-dose single acquisition 40 s after IV administration of 1.5 mL/kg iodinated contrast (concentration 320 mg Iodine/mL, flow rate 3 mL/s). A persistent left SVC was depicted, with drainage in the left atrium and resulting right-to-left shunt; a right SVC was consistently present, draining to the right atrium (Figure 1a,b). A bridging vein was depicted, with a mean diameter of 7 mm. A comprehensive cardiological assessment with echocardiography was performed, but no other anomalies were found. He was successfully treated by transcatheter occlusion: through the left internal jugular vein a vascular occlusive device (Amplatzer Vascular Plug 2) was inserted in the left SVC. Angiographic images and pressure measurements before plug insertion revealed that blood from the left jugular vein flowed in both the left SVC and the bridging vein, because of a gradient from left SVC to right SVC. After plug deployment, complete occlusion of the left SVC was obtained with normalization of the oxygen saturation. The dimensions of the bridging vein were not concerning for causing elevated left jugular pressure; vein stenting was not performed. CT images two months after plug deployment confirmed regular enlargement of the bridging vein, with mean diameter of 9 mm (Figure 2a,b). He is currently at six months follow up, with no symptoms, and practicing sport four times a week (tennis).

## 3. Discussion

The presence of a left SVC is uncommon and can be associated with different clinical and imaging scenarios. During embryological life a symmetric thorax venous system is present, with both right and left superior vena cava (SVC); normally, the left SVC obliterates in the first weeks of development [6]. In 85–90% of cases, persistent left SVC drains into the right atrium through a dilated coronary sinus, without blood shunts [6]; if the coronary sinus is partially unroofed, a small right-to-left shunt can occur. In the remaining 10–15% of cases, developmental arrest when the coronary sinus is still absent, the left SVC drains into the left atrium roof, causing a significant right-to-left shunt [5,7].

Left SVC draining into the right atrium is generally not associated with clinical manifestations; it is often discovered incidentally in adulthood during echocardiography or CT performed for other reasons [7]. It should be emphasized that left SVC draining into the right atrium increases the risk of complications during endovascular procedures: caution should be adopted during central line insertion or Swan-Ganz catheterization, because a catheter in the coronary sinus can cause angina or perforation; during pacemaker implantation the electrode should not pass through the SVC, because of the tortuosity of its last part resulting in instability [3,4]. 

Conversely, persistent left SVC with drainage in the left atrium can be related to relevant hemodynamic implications, allowing deoxygenated venous blood to bypass the lungs and return to the body [3]. Patients are usually school age children; they present increased risk of heart failure, intracerebral abscesses, disseminated infection and embolic cerebrovascular stroke [7,8,9]. Diagnosis could be done with echocardiography, but magnetic resonance (MR) imaging or low-dose CT with dedicated protocols are recommended to confirm the condition [8]. Surgical or transcatheter correction should be performed in patients with large shunt or clinically relevant symptoms; additional congenital heart disease and cardiac rhythm disorder should also be investigated and excluded [4,7]. In some cases, congenitally displaced or malformed sinus nodes or AV conduction systems have been observed [1]. After the treatment it is indicated to perform CT or MR to confirm left SVC occlusion and patency of right SVC; subsequently follow-up can be done with annual echocardiography [7]. Do not forget that other congenital vascular conditions can cause cyanosis in children, with varying degrees of severity [10]; it is often necessary to contact referral centers with high experience in diagnosis and treatment.

In conclusion, persistent left SVC is a rare vascular anomaly, often asymptomatic and incidentally detected. It can determinate right-to-left shunt with significant clinical implications; a broad spectrum of clinicians should be aware, in particular pediatricians, radiologists, cardiologists and cardiothoracic surgeons.
